# Presentation and outcomes of patients with clinically T1‐2, N0 parotid mucoepidermoid carcinoma: The roles of elective neck dissection and adjuvant radiotherapy

**DOI:** 10.1002/hed.27128

**Published:** 2022-06-22

**Authors:** Zaid Al‐Qurayshi, Christopher Blake Sullivan, Derek B. Allison, Marisa R. Buchakjian

**Affiliations:** ^1^ Department of Otolaryngology – Head & Neck Surgery University of Iowa Hospitals and Clinics Iowa City Iowa; ^2^ Head and Neck Institute Cleveland Clinic Foundation Cleveland Ohio USA; ^3^ Department of Pathology & Laboratory Medicine University of Kentucky College of Medicine Lexington Kentucky USA

**Keywords:** epidemiology, facial nerve, mucoepidermoid carcinoma, neck dissection, parotidectomy, radiotherapy, survival

## Abstract

**Objectives:**

Examine the role of elective neck dissection (END) and adjuvant radiotherapy (RT) in early‐stage clinically N0 parotid mucoepidermoid carcinoma (MEC).

**Methods:**

The study is a retrospective analysis of the National Cancer Database, 2004–2016. The study population included adult patients with MEC who underwent parotidectomy.

**Results:**

A total of 1233 patients were included. Histopathology demonstrated well, moderately, and poorly differentiated MEC 47.12%, 39.98%, and 12.90% of the time, respectively. END was performed in 78.67% of patients, resulting in nodal upstaging in 4.43% and identification of extracapsular extension (ECE) in 0.72%. RT was utilized in 67.33% of patients with advanced pathological features. Neither END nor RT improved overall survival separately (*p* < 0.05) or combined (adjusted HR: 1.19, 95%CI: 0.52, 2.70, *p* = 0.68).

**Conclusion:**

This study provides an epidemiological perspective regarding patients with clinically T1‐2, N0 MEC. There was no observed survival advantage with END and RT.

## 1 INTRODUCTION

Mucoepidermoid carcinoma (MEC) is the most common malignant neoplasm of major and minor salivary glands and occurs most often in the parotid gland.^1^ Although MEC comprises less than 1% of all malignancies, it accounts for almost 30% of malignant salivary gland neoplasms.^2^, ^3^ The incidence of MEC is highest in women between the third and fifth decade of life.^3^, ^4^

MEC tumors are composed of mucin‐secreting, intermediate, and epidermoid cells.^1^, ^5^ MEC may show variably cystic and solid growth patterns and frequently contains a tumor‐associated lymphoid proliferations around the invading edges.^4^ There are several well‐characterized histologic variants, including oncocytic, clear cell, sclerosing, solid, Warthin‐like, and others. Despite this morphologic diversity, most MECs harbor a *MAML2* gene fusions, which is specific to MEC and often helpful diagnostically.^6^, ^7^, ^8^ There is no universally accepted grading system, and, as a result, multiple grading systems exist. However, most grading systems are three‐tiered, with most tumors stratified from low, intermediate, or high grade based on the presence of various histopathologic features, including growth pattern (cystic versus solid, well‐circumscribed versus infiltrative), mitotic activity, necrosis, perineural invasion, lymphovascular invasion, and nuclear pleomorphism, among others.^9^ Lower grade tumors are often more cystic and well‐circumscribed, while higher grade tumors tend to be more solid and infiltrative with the aggressive aforementioned features.^4^

The 5‐year overall survival (OS) of MEC is 70.2%.^10^ Factors associated with poor survival include older age, increasing comorbidities, histologic grade, advanced stage tumors, and positive surgical margins.^10^, ^11^ Surgical excision is the primary treatment modality for MEC. However, the role of an elective neck dissection (END) in early‐stage tumors with clinically negative necks, including no lymphadenopathy on physical examination or diagnostic imaging, is variable given the lack of randomized clinical trials and treatment guidelines. END is routinely performed in patients with locoregional cervical nodal metastasis or high‐grade lesions.^4^ Additionally, the role of adjuvant radiotherapy (RT) and/or chemotherapy for MEC is not completely understood and tends to be physician‐ or institution‐dependent, with chemotherapy being used in the palliative setting for advanced or recurrent disease.^12^, ^13^ The objective of the current study is to examine the epidemiological characteristics and treatment patterns among patients with clinically T1‐2, N0 MEC of the parotid gland.

## 2 MATERIALS AND METHOD

This study is a retrospective cohort analysis utilizing the National Cancer Database (NCDB) from 2004 to 2016.^14^ The NCDB is a joint program between the American College of Surgeons (ACS) and the American Cancer Society that contains data collected from facilities accredited by the Commission on Cancer.^14^ The NCDB, established in 1989, is a nationwide, facility‐based, comprehensive clinical surveillance resource with an oncology data set that currently captures 70% of all newly diagnosed malignancies in the United States annually.^14^ The NCDB is de‐identified data that does not meet the criteria of human subject research and does not meet the criteria of review by the University of Iowa Institutional Review Board.^14^ The ACS and Commission on Cancer (CoC) have not verified and are not responsible for the statistical validity of the data analysis or the conclusions derived by the authors.

The objective of the study is to describe the presentation and management of patients with T1‐2, N0 parotid MEC with a focus on the practice patterns of elective neck dissection and adjuvant radiotherapy, as well as their impact on OS.

The study population included adult patients (age ≥18 years) who were diagnosed with clinically T1‐2, N0 parotid MEC. MEC was identified based on the International Classification of Diseases for Oncology third edition (ICD‐O‐3: 8430). The study population was classified based on grade of tumor differentiation and based on the absence or presence of one or more advanced pathological features, including poorly differentiated tumor, pathological T3‐4a, positive lymph node(s), positive surgical margins, lymphovascular invasion (LVI), and extracapsular extension (ECE). It should be noted that LVI was available only for the years 2010–2016. The data were checked for completeness of other study parameters and patients with missing values were excluded.

Independent variables of interest included the following: age, sex, Charlson/Deyo score as reported in the NCDB,^14^ laterality, type of parotidectomy (superficial vs. total), and whether the facial nerve was preserved or sacrificed.

Chi‐square was used to assess for the association of each independent factor with tumor grade, probability of undergoing elective neck dissection, and probability of utilizing adjuvant radiotherapy. Variables that demonstrated significant association were included in the multivariate logistic regression models that were used to calculate odds ratio (OR) and 95% confidence interval (95%CI). Log‐rank test was performed to assess the association of each independent variable with OS. Variables that demonstrated significant association were included in the multivariate Cox hazard ratio (HR) models. Cox hazard ratio was also used to examine and control for time‐interaction terms of each independent factor. Significant level was set as (*α* = 0.05). All statistical analyses were performed using SAS 9.4 (SAS Institute Inc., Cary, NC).

## 3 RESULTS

A total of 1233 patients were included. Characteristics of the study population are listed in Table 1. The mean age of patients at the time of diagnosis was 55.80 ± 15.90 years, 61.15% were female, and 80.45% had a Charlson/Deyo score of 0. Median follow‐up time was 57.69 months (interquartile range: 35.71–91.30 months).

**TABLE 1:** Descriptive statistics of the study population of patients with clinically T1‐2, N0 parotid mucoepidermoid carcinoma who underwent surgical resection. National Cancer Database, 2004–2016

	Study population, *N* = 1,233 (%)^a^	Grade (%)^a^			*p*‐value^b^
		Well differentiated, *n* = 581	Moderately differentiated, *n* = 493	Poorly differentiated, *n* = 159	
Age (year)					
<65	845 (68.53)	417 (71.77)	360 (73.02)	68 (42.77)	<0.001
≥65	388 (31.47)	164 (28.23)	133 (26.98)	91 (57.23)	
Sex					
Male	479 (38.85)	209 (35.97)	177 (35.9)	93 (58.49)	<0.001
Female	754 (61.15)	372 (64.03)	316 (64.1)	66 (41.51)	
Charlson/Deyo score					
0	992 (80.45)	461 (79.35)	405 (82.15)	126 (79.25)	0.48
1	183 (14.84)	88 (15.15)	67 (13.59)	(12.03–24.44)^c^	
≥2	58 (4.7)	32 (5.51)	21 (4.26)	(01.03–07.19)^c^	
Laterality					
Right parotid gland	626 (50.77)	288 (49.57)	258 (52.33)	80 (50.31)	0.66
Left parotid gland	607 (49.23)	293 (50.43)	235 (47.67)	79 (49.69)	
Surgery					
Superficial parotidectomy	717 (58.15)	348 (59.9)	298 (60.45)	71 (44.65)	0.001
Total parotidectomy	516 (41.85)	233 (40.1)	195 (39.55)	88 (55.35)	
Facial nerve					
Preserved	921 (74.7)	439 (75.56)	383 (77.69)	99 (62.26)	<0.001
Sacrificed	312 (25.3)	142 (24.44)	110 (22.31)	60 (37.74)	
Elective neck dissection					
Not performed	263 (21.33)	124 (21.34)	108 (21.91)	31 (19.5)	0.81
Performed	970 (78.67)	457 (78.66)	385 (78.09)	128 (80.5)	
Pathological T					
1	784 (63.58)	406 (69.88)	330 (66.94)	48 (30.19)	<0.001
2	354 (28.71)	154 (26.51)	121 (24.54)	79 (49.69)	
3	68 (5.52)	(01.85–04.85)^c^	(04.48–09.04)^c^	18 (11.32)	
4a	27 (2.19)	(00.11–01.50)^c^	(00.98–03.70)^c^	14 (8.81)	
Outcome of elective neck dissection					
Pathological N0	920 (94.85)	448 (98.03)	363 (94.29)	109 (85.16)	<0.001
Pathological N+ without ECE	(03.23–05.93)^c^	(00.76–03.42)^c^	(03.20–07.91)^c^	(06.71–18.59)^c^	
Pathological N+ with ECE	(00.29–01.48)^c^	(00.01–01.21)^c^	(00.06–01.86)^c^	(00.86–07.81)^c^	
Surgical margins					
Negative	998 (80.94)	499 (85.89)	389 (78.9)	110 (69.18)	<0.001
Positive	235 (19.06)	82 (14.11)	104 (21.1)	49 (30.82)	
Lymphovascular invasion^d^					
Not present	745 (96.63)	(97.67–99.83)^c^	(94.90–98.86)^c^	77 (83.7)	<0.001
Present	26 (3.37)	(00.17–02.33)^c^	(01.14–05.10)^c^	15 (16.3)	
T3‐4a, LVI, N+, ECE, and/or positive surgical margins					
Not present	905 (73.40)	479 (82.44)	350 (70.99)	76 (47.80)	<0.001
Present	328 (26.60)	102 (17.56)	143 (29.01)	83 (52.20)	
Adjuvant radiotherapy					
Not performed	793 (64.31)	452 (77.8)	308 (62.47)	33 (20.75)	<0.001
Performed	440 (35.69)	129 (22.2)	185 (37.53)	126 (79.25)	

Abbreviations: ECE, extracapsular extension; LVI, lymphovascular invasion.

^a^ Percentage values add‐up vertically and may not add up to 100% due to rounding.

^b^ Chi‐square test.

^c^ This range represents 95% confidence interval. Data‐using‐agreement prohibits reporting exact number of subjects for variables with less than 10 subjects with the event so it was substituted with 95% confidence interval to convey an estimate.

^d^ Lymphovascular invasion variable is missing for 462 subjected because it is only available for the years 2010–2016.

**FIGURE 1:**
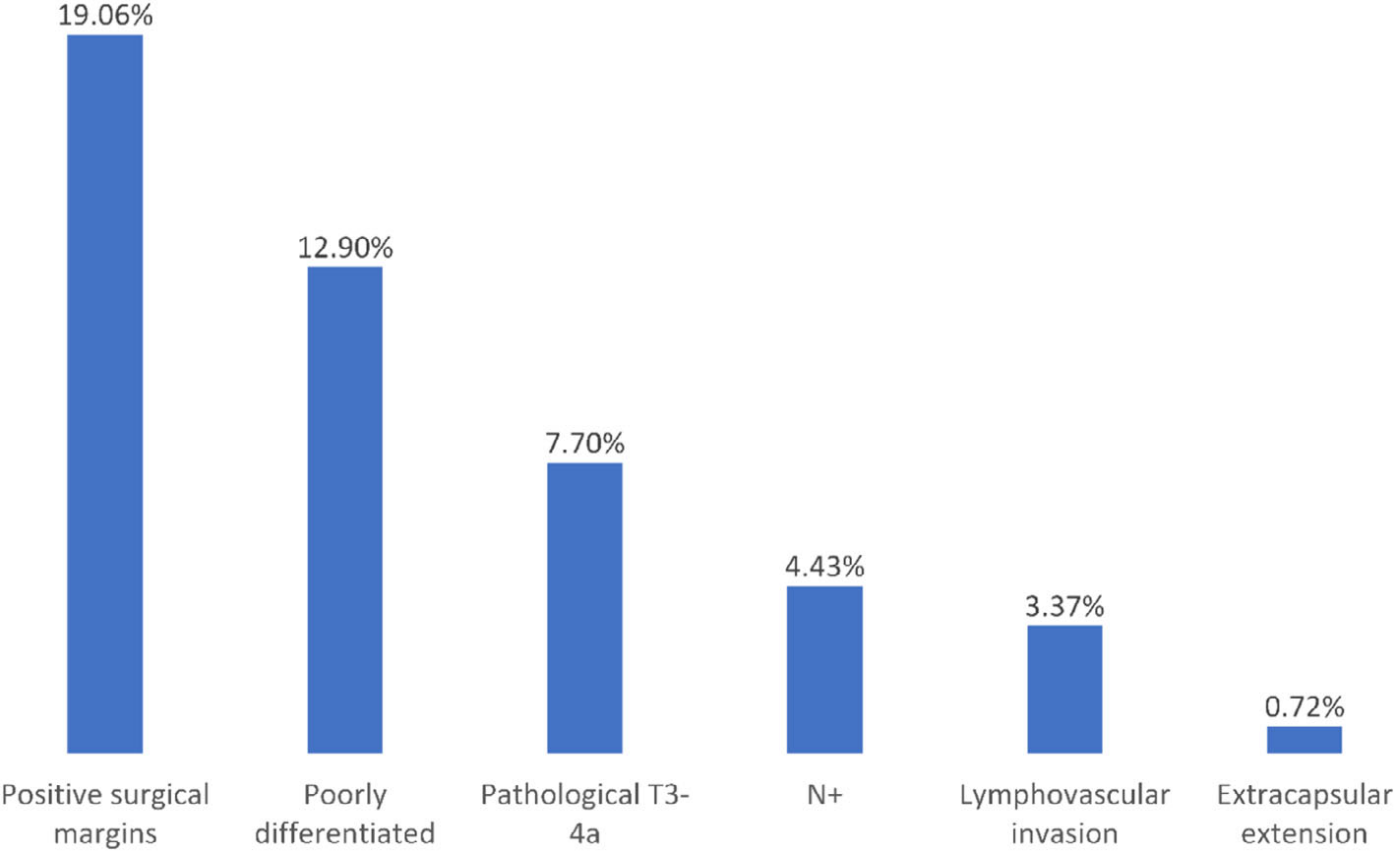
Probability of advanced pathological features in patients with clinically T1‐2, N0 parotid mucoepidermoid carcinoma who underwent surgical resection [Color figure can be viewed at wileyonlinelibrary.com]

The majority of patients underwent a superficial parotidectomy (58.15%), elective neck dissection (78.67%), and the preservation of the facial nerve (74.70%). Facial nerve sacrifice was significantly higher in patients who underwent total parotidectomy compared to superficial parotidectomy (30.81% vs. 21.34%, *p* < 0.001). It is worth noting that the NCBD does not specify if the sacrifice of the facial nerve involved the main trunk or a branch of the facial nerve. The prevalence of well, moderately, and poorly differentiated MEC was 47.12%, 39.98%, and 12.90%, respectively. Surgical margins were negative in 80.94% of patients, and lymphovascular invasion was absent in 96.63% of patients. T classification was upstaged in 11.52% of the patients (Figure 1).

**FIGURE 2:**
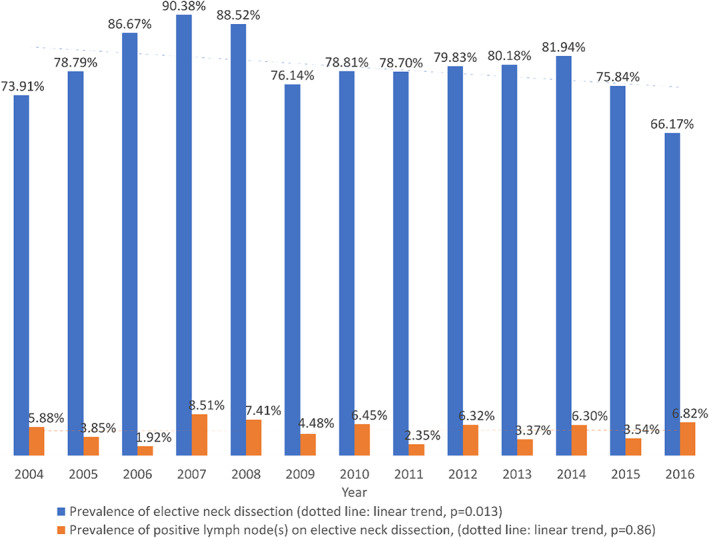
Prevalence of elective neck dissection and positive lymph node(s) in relation to the study period in patients with clinically T1‐2, N0 parotid mucoepidermoid carcinoma who underwent surgical resection [Color figure can be viewed at wileyonlinelibrary.com]

Among patients who underwent elective neck dissection, 94.9% of patients were pathologically N0. The performance of elective neck dissection tended to decrease over the study period (*p* = 0.013) (Figure 2). Elective neck dissection was not associated in the univariate analysis with any of the demographic or clinical factors so multivariate logistic regression for the probability of performing elective neck dissection was not performed. In patients who underwent neck dissection, the odds of identifying positive lymph node(s) was significantly associated in the multivariate model with poorly/moderately differentiated tumor, pathological T3‐4, positive surgical margins, and presence of lymphovascular invasion (Table 2).

**TABLE 2:** Multivariate analysis of factors associated with likelihood of identifying positive lymph node(s) on neck dissection of patients with clinically T1‐2, N0 mucoepidermoid carcinoma

	% positive LN(s) on pathology	OR^a^	95%CI	*p*‐value
Grade				
Well differentiated	1.97	Reference		
Moderately differentiated	5.71	3.84	1.23, 12.01	0.021
Poorly differentiated	14.84	5.08	1.40, 18.48	0.014
Pathological T				
1–2	3.92	Reference		
3–4	19.23	3.05	1.16, 7.99	0.023
Surgical margins				
Negative	3.77	Reference		
Positive	11.43	2.63	1.13, 6.12	0.025
Lymphovascular invasion				
Not present	3.62	Reference		
Present	22.73	4.53	1.36, 15.05	0.014

Abbreviations: CI, confidence interval; ECE, extracapsular extension; LN, lymph node; OR, odds ratio.

^a^ Multivariate logistic regression model included all the variables listed in the table except lymphovascular invasion as it was analyzed in a separate model that also included the other variables because lymphovascular invasion was only available for years 2010–2016.

**FIGURE 3:**
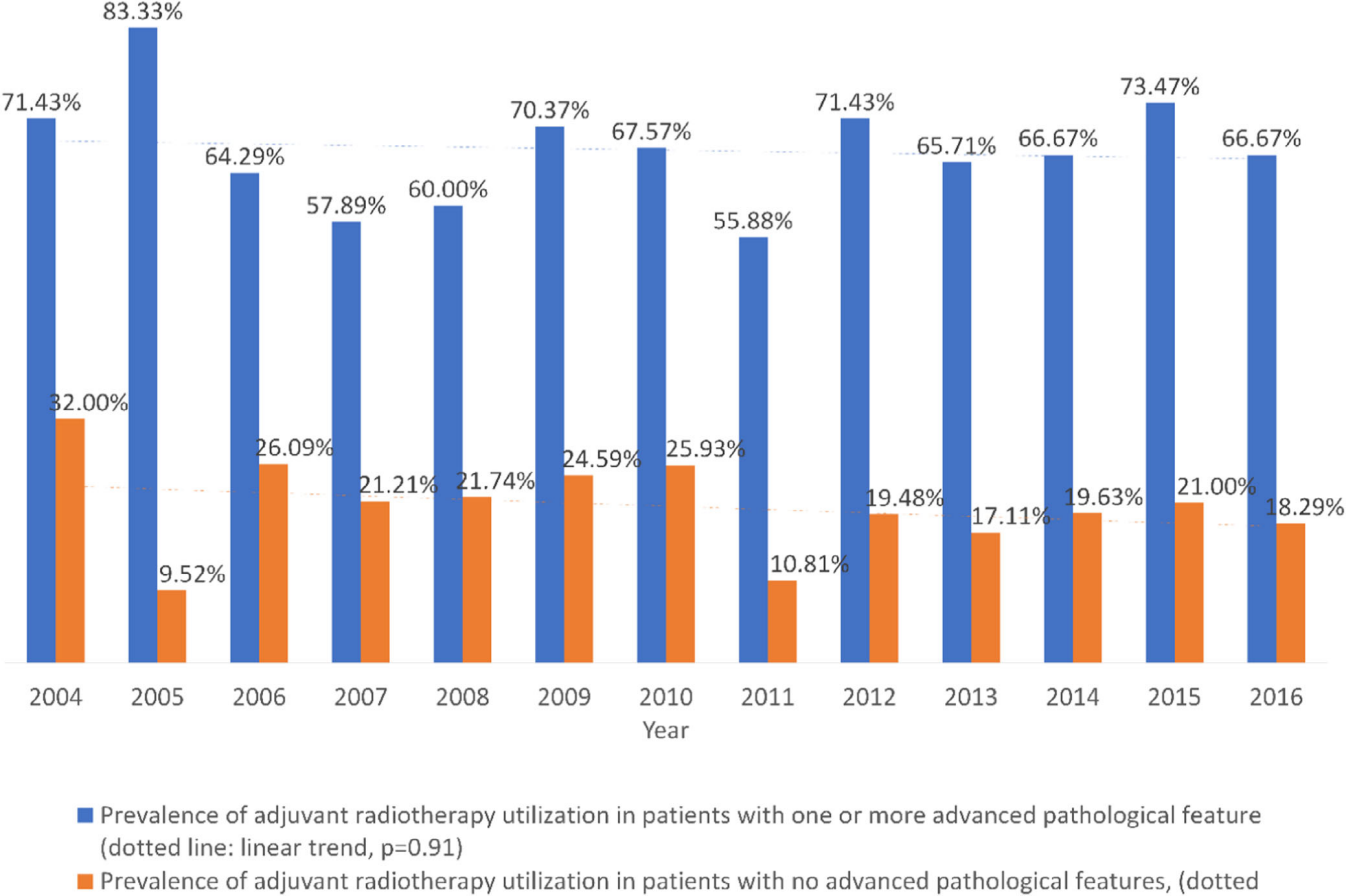
Prevalence of adjuvant radiotherapy utilization in relation to the study period and the presence of advanced pathological features (poorly differentiated, T3‐4a, lymphovascular invasion, extracapsular extension, positive surgical margins) in patients with clinically T1‐2, N0 parotid mucoepidermoid carcinoma who underwent surgical resection [Color figure can be viewed at wileyonlinelibrary.com]

RT was performed in 35.7% of patients. RT annual utilization in relation to MEC pathological features are demonstrated in Figure 3. There was no significant trend in utilization throughout the study period. Patient factors that were associated with higher likelihood of RT utilization included poorly differentiated tumors (OR 12.16, 95%CI 6.69–22.12; *p* < 0.001), pathological T3 or T4 tumors (OR 2.45, 95%CI 1.29–4.68; *p* = 0.006), and positive surgical margins (OR 3.68, 95%CI 2.42–5.59; *p* < 0.001) (Table 3).

**TABLE 3:** Multivariate analysis of factors associated with likelihood of utilizing adjuvant radiotherapy in patients with clinically T1‐2, N0 mucoepidermoid carcinoma who underwent surgical resection

	% adjuvant radiotherapy	OR^a^	95%CI	*p*‐value
Surgery				
Superficial parotidectomy	31.66	Reference		
Total parotidectomy	41.28	1.03	0.72, 1.46	0.89
Grade				
Well differentiated	22.20	Reference		
Moderately differentiated	37.53	1.94	1.35, 2.79	<0.001
Poorly differentiated	79.25	12.16	6.69, 22.12	<0.001
Pathological T				
1–2	32.78	Reference		
3–4	70.53	2.45	1.29, 4.68	0.006
Elective neck dissection outcomes				
Elective neck dissection not performed	34.22	0.67	0.43, 1.03	0.07
Pathological N0	33.80	Reference		
Pathological N+ without ECE	76.74	4.94	1.54, 15.81	0.007
Pathological N+ with ECE	85.71	0.24	0.01, 11.57	0.47
Surgical margins				
Negative	28.56	Reference		
Positive	65.96	3.68	2.42, 5.59	<0.001
Lymphovascular invasion				
Not present	33.02	Reference		
Present	69.23	1.44	0.52, 4.02	0.48

Abbreviations: CI, confidence interval; ECE, extracapsular extension; OR, odds ratio.

^a^ Multivariate logistic regression model included all the variables listed in the table except lymphovascular invasion as it was analyzed in a separate model that also included the other variables because lymphovascular invasion was only available for year 2010–2016.

**FIGURE 4:**
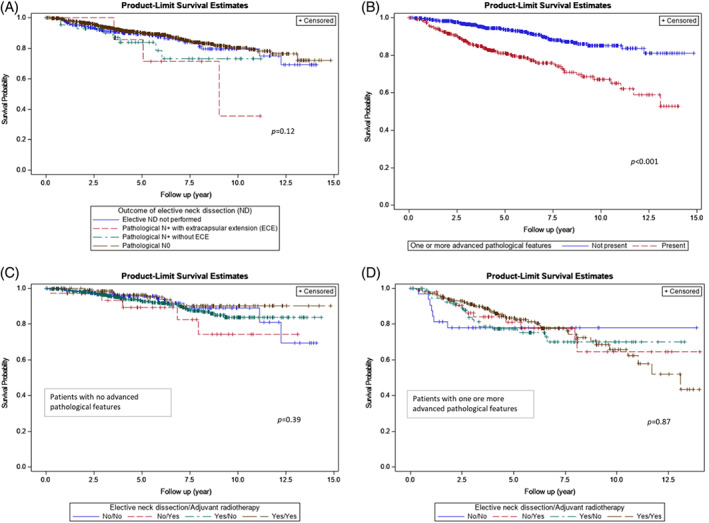
Overall survival of patients presenting with clinically T1‐2, N0 parotid mucoepidermoid carcinoma based on (A) elective neck dissection outcome, (B) absence or presence of one or more advanced pathological features (poorly differentiated, T3‐4a, positive lymph node(s), positive surgical margins, lymphovascular invasion, extracapsular extension), (C) whether elective neck dissection and adjuvant radiotherapy were utilized in patients with no advanced pathological features, (D) whether elective neck dissection and adjuvant radiotherapy were utilized in patent with one or more advanced pathological features [Color figure can be viewed at wileyonlinelibrary.com]

Among patients with no adverse pathological features, age 65 years and older (adjusted HR 4.58, 95%CI 2.77–7.59; *p* < 0.001), male sex (adjusted HR 2.22, 95%CI 1.36–3.60; *p* = 0.001), and total parotidectomy (adjusted HR 4.06, 95%CI 1.61–10.26; *p* = 0.003) were associated with worse OS (Table 4). With respect to patients with one or more advanced pathological features, age 65 years and older (adjusted HR 6.13, 95%CI 3.59–10.45; *p* < 0.001), Charlson/Deyo score ≥2 (adjusted HR 2.96, 95%CI 1.24–7.08; *p* = 0.014), and total parotidectomy (adjusted HR 2.26, 95%CI 1.07–4.78; *p* = 0.34) were associated with worse OS. Comparing the two groups, OS was worse among patients with one or more advanced pathological feature (Figure 4). In analyzing the addition of an elective neck dissection, adjuvant radiotherapy, or both in combination, there was no effect observed on OS.

**TABLE 4:** Five‐year overall survival probability and adjusted hazard ratio for patients presented with clinically T1‐2, N0 parotid mucoepidermoid carcinoma in relation to demographic and clinical factors classified based on pathological features

Group	Factor	% 5‐year overall survival	HR^a^	95%CI	*p*‐value
No advanced pathological features	Age (year)				
	<65	97.53	Reference		
	≥65	86.15	4.58	2.77, 7.59	<0.001
	Sex				
	Male	90.79	2.22	1.36, 3.60	0.001
	Female	95.99	Reference		
	Charlson/Deyo score				
	0	94.39	Reference		
	1	92.00	1.33	0.75, 2.33	0.33
	≥2	90.81	1.46	0.45, 4.75	0.53
	Surgery				
	Superficial parotidectomy	96.02	Reference		
	Total parotidectomy	90.88	4.06	1.61, 10.26	0.003
	Facial nerve				
	Preserved	94.82	Reference		
	Sacrificed	91.90	1.56	0.92,2.65	0.10
	Elective neck dissection/adjuvant radiotherapy performed				
	No/no	95.61	1.54	0.60, 3.90	0.37
	Yes/no	93.85	1.46	0.69, 3.12	0.32
	No/yes	89.35	2.42	0.78, 7.47	0.13
	Yes/yes	97.38	Reference		
One or more advanced pathological features (poorly differentiated, T3‐4A, positive lymph node(s), positive surgical margins, lymphovascular invasion, extracapsular extension)	Age (year)				
	<65	93.09	Reference		
	≥65	65.12	6.13	3.59, 10.45	<0.001
	Sex				
	Male	76.78	1.24	0.78, 1.96	0.37
	Female	86.60	Reference		
	Charlson/Deyo score				
	0	85.66	Reference		
	1	67.58	1.47	0.86, 2.50	0.16
	≥2	77.00	2.96	1.24, 7.08	0.014
	Surgery				
	Superficial parotidectomy	85.90	Reference		
	Total parotidectomy	79.57	2.26	1.07, 4.78	0.034
	Facial nerve				
	Preserved	85.75	Reference		
	Sacrificed	79.88	1.20	0.75, 1.93	0.44
	Elective neck dissection/adjuvant radiotherapy performed				
	No/no	87.50	1.19	0.52, 2.70	0.68
	Yes/no	81.32	1.41	0.82, 2.43	0.21
	No/yes	80.97	0.93	0.48, 1.77	0.82
	Yes/yes	86.00	Reference		

Abbreviations: CI, confidence interval; HR, hazard ratio.

^a^ Multivariate Cox hazard ratio model includes all the variables in the table.

## 4 DISCUSSION

MEC comprises 30% of all major salivary gland tumors, but the incidence of MEC of the parotid gland is only 2.3 per 1000000.^11^ These tumors are relatively rare with considerable heterogeneity and are characterized by multiple grading systems, which makes identification of risk factors and definitive treatment recommendations elusive. This study provides an understanding of epidemiological characteristics, treatment factors, and treatment patterns of patients with T1‐2, N0 MEC of the parotid gland. Importantly, we found that OS was not improved with END and/or RT.

Clinical staging relies on physical examination and preoperative imaging, including computed tomography and in some instances magnetic resonance imaging. Disparities exist between clinical and pathological staging for salivary gland MEC. Similar to other head and neck cancers, the discrepancy may be due to clinical staging failure or, perhaps, tumor progression prior to surgery.^15^ The current study provides useful information about pathological upstaging for T1‐2, N0 MEC of the parotid gland. T classification was upstaged in 11.52% of the patients after surgery; 5.52% to T3 and 2.19% to T4a. Patients who were upstaged to T3 or T4 classification were more likely to undergo RT. N classification was upstaged in 4.43% of patients who underwent END. Having accurate clinicopathological staging is important in deciding treatment. In patients with clinically N0 and underwent neck dissection, predictors of identifying positive lymph node(s) was associated with poorly/moderately differentiated tumors, pathological T3‐4 tumors, positive surgical margins, and presence of lymphovascular invasion. Those predictors are on histopathological examination. We did not identify demographic predictors that can be considered in the preoperative setting to identify patients at high‐risk of occult nodal disease. It should be noted that the analysis is limited in terms of identifying preoperative factors because the NCDB does not include detailed preoperative data such as information regarding preoperative physical examination, imaging, or laboratory studies.

The type of parotidectomy for early salivary gland cancers, either extracapsular or partial with or without facial nerve dissection, is a topic of debate. Some advocates for a partial parotidectomy when the tumor is cancerous due to the risk of indistinct or infiltrative margins.^16^ However, a retrospective series of 30 patients who had a preoperative diagnosis of a benign parotid gland tumor, but were found to have a malignancy on histopathology after an extracapsular dissection, did not have adverse outcomes on survival.^17^ The aforementioned study was a small case series, and the study may not be adequately powered to fully address this question. In a study by Mantsopoulos et al. that included nine patients with primary low‐grade parotid malignant neoplasms who underwent extracapsular dissection, they demonstrated 5‐year disease‐specific survival of 100%.^18^ In a systematic review of 2202 patients by Guntinas‐Lichius et al., they identified intraparotid metastasis as a negative prognostic factor for primary parotid gland cancer.^19^ There is still a controversy regarding extent of parotidectomy (total vs. superficial) for tumors confined to the superficial lobe with superficial intraparotid lymph node metastasis and cervical metastasis without deep lobe intraparotid metastasis.^19^ Future studies are warranted to better delineate the role of intraparotid lymph node metastasis.

A parotidectomy, whether partial or total, is standard of care for MEC, but management of the neck is physician and institutionally dependent. Due to significant heterogeneity among salivary gland cancers, including MEC, the management of the N0 neck is controversial. Many factors, including tumor histology, adverse features, and patient morbidity, among many others, influence the decision to perform an END. Management decisions regarding MEC of the parotid are often based on small retrospective studies.^20^ Armstrong et al. reviewed 474 patients with major salivary gland cancer and found 14% of MEC patients had clinically occult, cervical nodal metastasis.^21^ The strongest predictor of occult metastasis was high grade tumors.^21^ Similarly, Lau et al. reviewed 119 patients with salivary gland cancer of the head and neck and found occult, cervical nodal metastasis in 35% of high‐grade MEC patients compared to 10 and 0% for intermediate and low‐grade MEC, respectively.^22^ These studies had concordant findings that patients with high‐grade MEC would likely benefit from an END. However, in the present much larger study, patients with and without adverse pathological features did not have a significantly different 5‐year OS compared to patients treated with an END.

Treatment patterns are often based of the National Comprehensive Cancer Network's salivary gland cancer guidelines. For early T classification (T1, T2) salivary gland cancers, consideration of RT is warranted with tumor spillage, perineural invasion, or intermediate or high‐grade tumors.^23^ Given the lack of consensus for histologic grading of salivary gland MEC, there is potential for interobserver variability in RT treatment recommendations. In the present study, patients with tumors that were pathologically upstaged or had positive surgical margins were more likely to undergo RT. However, RT was not associated with improved OS. The role of RT in MEC of the parotid gland is not clearly defined and remains a controversial topic.^24^ In an analysis of 47 patients with early stage MEC of the salivary glands and a known favorable genetic mutation, Okumura et al. reported a 60 month median survival rate of 100% after surgical resection without RT.^25^ While OS benefit was not seen with RT, caution must be employed. Direct comparison of surgery alone versus surgery with RT has the potential of selection bias as the patients who underwent RT are more likely to have advanced disease. Additionally, the NCDB does not code for disease‐specific survival. Future prospective clinical trials are warranted to validate the present findings.

The role of facial nerve function is a key consideration for any patient undergoing a parotidectomy. Pre‐operative facial nerve dysfunction is believed to be the result of direct tumor invasion, perineural invasion, or interruption of the blood supply to the nerve.^26^ Weakness of the nerve preoperatively is a known predictor of facial nerve sacrifice during parotidectomy.^26^ Proper oncologic management should not be prevented by preserving the facial nerve. Facial nerve paralysis is most common with undifferentiated carcinoma (24%) compared to MEC (9%).^27^ We found that 75% of patients with early stage MEC were able to have their facial nerve preserved. It is worth noting that the NCBD does not specify if the sacrifice of the facial nerve involved the main trunk or a branch of the facial nerve. A study of 75 patients with parotid malignancy found that they were able to spare the nerve in 60% of patients.^26^ Facial nerve sacrifice was not associated with worse OS in patients with no adverse features or in patients with adverse features. The decision to sacrifice the nerve has significant functional and cosmetic consequences, and this data provides physicians with relevant information to help counsel patients preoperatively about the potential for facial nerve preservation.

There are inherent and multiple limitations to the present study. Causality cannot be established as this is a retrospective cohort study. There is a lack of information regarding preoperative imaging studies and operative details. The database does not have information regarding the decision‐making process and reasons behind choosing certain management. Furthermore, a lack of centralized pathologic review and reassessment of adverse pathologic features may result in some inconsistent results; however, data from such a large number of facilities may represent more generalizable results. Next, database entry can lead to patient coding and classification errors, and the study has potential for selection bias given patients are from CoC accredited hospitals.^14^ While the database has a wide variety of coding variables, the NCDB does not describe key clinical details such as the extent of END performed, number of procedures, and detailed procedural findings. This lack of treatment data limits the ability to identify all factors influencing treatment outcomes. Over the length of this retrospective series, grading of histologic subtypes has been changed, which would alter tumor diagnosis retrospectively and lead to an inherent limitation. Even with the limitations, the present study provides meaningful epidemiological information and treatment outcomes about patients with T1‐2, N0 MEC of the parotid gland.

## 5 CONCLUSION

In this large analysis of patients with T1‐2, N0 MEC of the parotid gland, we found that T classification was upstaged 11.52% of the time, and the facial nerve was preserved in 74.7% of patients. We found that END is routinely performed in T1‐2, N0 MEC of the parotid gland, but there was no observed difference in OS. Additionally, utilization of adjuvant RT did not appear to improve OS. These findings provide meaningful information to help physicians counsel patients preoperatively with early stage MEC of the parotid gland.

## CONFLICT OF INTEREST

The authors declare that there is no conflict of interest that could be perceived as prejudicing the impartiality of the research reported.

## Data Availability

The data‐using agreement prohibits sharing data. The data are available by application to the sponsoring agency, the American College of Surgeons.
